# Acute Periprosthetic Hip Joint Infection Caused by Multidrug-Resistant Acinetobacter Baumannii: Is Debridement, Antibiotics, Irrigation, and Implant Retention a Viable Treatment Option?

**DOI:** 10.7759/cureus.13090

**Published:** 2021-02-03

**Authors:** Angelo V Vasiliadis, Frideriki Poutoglidou, Vasiliki Chatziravdeli, Dimitrios Metaxiotis, Anastasios Beletsiotis

**Affiliations:** 1 Orthopaedics, 2nd Orthopaedic Department, General Hospital of Thessaloniki “Papageorgiou”, Thessaloniki, GRC; 2 Department of Anatomy and Surgical Anatomy, School of Medicine, Faculty of Health Sciences, Aristotle University of Thessaloniki, Thessaloniki, GRC; 3 Orthopaedics, General Hospital of Thessaloniki “Papageorgiou”, Thessaloniki, GRC

**Keywords:** periprosthetic joint infection, acinetobacter baumannii, dair, arthroplasty, antibiotic treatment

## Abstract

In this study, we aimed to investigate the effectiveness of debridement, antibiotics, irrigation, and implant retention (DAIR) in periprosthetic hip joint infection caused by multidrug-resistant (MDR) *Acinetobacter baumannii (A. baumannii)*.

From July 2019 to June 2020, we retrospectively reviewed all patients treated for periprosthetic hip joint infections caused by MDR *A. baumannii *at our institution. The diagnosis of periprosthetic joint infection (PJI) was established based on the Musculoskeletal Infection Society (MSIS) 2018 criteria. The Charlson Comorbidity Index (CCI) was used to estimate the risk of mortality. The patients were followed up for over a year, until their death, or loss to follow-up.

Four patients (three females and one male), with a mean age of 68 years, were included in the study. *A. baumannii *exhibited resistance to fluoroquinolones in all cases. All patients were treated with the DAIR procedure followed by intravenous tigecycline and colistin combination treatment. Prosthesis retention with good functional results was achieved in two patients. One patient required resection arthroplasty and one patient died two months after the initial surgical treatment, yielding a success rate of 50% for the DAIR procedure.

Periprosthetic hip joint infection caused by MDR* A. baumannii* is one of the most demanding and challenging complications in orthopaedic practice. This case series suggests that the outcome of the DAIR is affected by a number of factors that are in a complex interplay. Our results indicate a limited success rate for the DAIR procedure in the treatment of a periprosthetic hip joint infection caused by MDR *A. baumannii*.

## Introduction

Periprosthetic joint infection (PJI) is a rare but major complication after hip replacement, with an incidence of 0.5-3%; it results in significant morbidity and mortality [1]. The risk of developing a PJI is associated with several potentially patient-related factors, such as sociodemographic characteristics, body mass index (BMI), and medical/surgical history [2]. Management of a PJI is challenging and usually requires complex treatment strategies in order to provide favorable outcomes related to patients’ quality of life [3]. For early postoperative PJIs (occurring within 30 days of implantation), debridement, antibiotics, irrigation, and implant retention (DAIR) is the conventional treatment option. The DAIR procedure should be performed promptly after the onset of the symptoms of infection [2]. Failure of the DAIR is related to existing preoperative parameters, such as the immunocompromised state in patients, persistent wound drainage, and specific bacterial pathogens isolated from the culture [4].

*Acinetobacter* species are aerobe gram-negative microorganisms that typically colonize the skin surface. Previous studies report a colonization rate of 25-40% and 75% among healthy individuals and hospitalized patients respectively. Moreover, the respiratory tract of patients is frequently colonized by *Acinetobacter* species during hospitalization [5]. Nosocomial infections caused by *Acinetobacter* species represent a significant clinical problem, with the overall incidence of PJI due to *Acinetobacter* species ranging from 0.6% to 1.2% according to recent literature [6,7]. Infections due to *Acinetobacter* *Baumannii* (*A. baumannii*) is indicative of severe illness and is related to a 30% higher risk of mortality [8]. In the present study, we retrospectively reviewed four cases of multidrug-resistant (MDR) *A. baumannii *PJI, in order to assess the efficacy of the DAIR procedure.

## Case presentation

We performed a single-center, retrospective case series study. The study was examined and approved by our hospital’s Institutional Review Board. Over a period of 12 months (from July 2019 to June 2020), patients over 18 years of age, with a periprosthetic hip joint infection caused by *A. baumannii*, and who underwent DAIR, were included in the study. Patient data were acquired from the department’s medical and surgical indices, as well as the microbiology database. A dedicated data collection form was developed for recording all relevant details. Patients were followed up for over a year, until their death, or loss to follow-up.

The diagnosis of PJI was established based on the Musculoskeletal Infection Society (MSIS) 2018 criteria and the International Consensus Meeting [9]. Therefore, the infection was defined as the presence of a sinus tract communicating with the articular cavity, or the isolation of the pathogen by intraoperative culture in two separate specimens obtained from the affected prosthetic joint, or the presence of at least three of the following minor criteria: i) elevated C-reactive protein (CRP) and erythrocyte sedimentation rate (ESR), ii) elevated synovial fluid white blood cell (WBC) or ++ change on leukocyte esterase test strip, iii) elevated synovial fluid polymorphonuclear neutrophil percentage (PMN%), iv) a positive histological analysis of the periprosthetic tissue, and v) a single positive synovial culture.

As previously described, PJI was defined as acute when symptom duration was less than three weeks or if the symptoms developed within the first four weeks after arthroplasty [3]. Meanwhile, the Charlson Comorbidity Index (CCI) adjusted for age was calculated. The CCI, an indicator of comorbidity in orthopaedic patients, is commonly used to predict prolonged hospitalization, complications, and mortality. Finally, patients were classified as having a good outcome or treatment failure. Treatment failure was defined based on patients meeting at least one of the following criteria: i) recurrence of the PJI due to *A. baumannii* strain or a different microorganism, ii) death due to a prosthesis-related infection, iii) clinical, laboratory, or radiological findings suggestive of a PJI at any time after the initial treatment, and iv) a need for resection arthroplasty.

Four patients (three females and one male with a mean age of 68 years, range: 64-74 years) were diagnosed with MDR *A. baumannii *PJI. Patients’ demographics data are presented in Table 1. One patient had a cemented bipolar hemiarthroplasty due to a previous femoral neck fracture, two patients had undergone primary total hip arthroplasty, and one patient had undergone revision total hip arthroplasty. Major comorbidities present were hypertension (n=3), hypothyroidism (n=2), atrial fibrillation (n=2), diabetes mellitus (n=1), and dyslipidemia (n=1). Main symptoms were pain (n=4), wound drainage (n=3), and fever (temperature of >37.5-39 ^o^C, n=2). The mean CCI was 51 (range: 21-77) (Table 1).

**Table 1:** Characteristics of four patients with periprosthetic hip joint infection caused by multidrug-resistant Acinetobacter Baumannii The detection limit of CRP was >0.8 mg/dL BMI: body mass index; CCI: Charlson Comorbidity Index; OA: osteoarthritis; THA: total hip arthroplasty; HA: hip arthroplasty; HTN: hypertension; DLP: dyslipidemia; HT: hypothyroidism; DM: diabetes mellitus; COPD: chronic obstructive pulmonary; AF: atrial fibrillation; GERD: gastroesophageal reflux disease; T: tigecycline; Co: colistin; V: vancomycin; Te: teicoplanin; Res. A: resection arthroplasty

Characteristics	Case 1	Case 2	Case 3	Case 4
Gender	Female	Female	Female	Male
Age, years	67	64	74	67
BMI, kg/m^2^	22.03	20.95	25.7	30.9
CCI, %	77	53	21	53
Indication	Aseptic loosening	Femoral neck fracture	Femoral neck fracture	Secondary OA
Arthroplasty	Revision THA	Primary THA	Bipolar HA	Primary THA
Comorbidities	HTN, DLP	HTN, HT, DM, COPD, anemia, depression	HTN, HT, AF, GERD	AF
Signs and symptoms				
Pain	Yes	Yes	Yes	Yes
Fever	No	Yes	No	Yes
Wound drainage	Yes	Yes	Yes	No
Laboratory findings				
WBC, x 10^3^/μL	7.46	6.22	7.23	6.3
CRP, mg/dL	2.96	23.8	3.08	16.3
ESR, mm/h	95	123	72	120
Culture sample (number)	synovial fluid, deep tissue (5)	synovial fluid, deep tissue, implant (5)	Synovial fluid, deep tissue (5)	Deep tissue, implant (5)
IA-DAIR, days	24	20	33	25
Antibiotics IV (days)	T, Co, V, Te (23)	T, Co, V (20)	T, Co, V (23)	T, Co, V, Te (28)
Surgical management	DAIR	DAIR (x 2), Res. A	DAIR	DAIR, Res. A
Failed treatment	No	Yes	No	Yes
Functional outcome	Independent life	Wheelchair user	Independent life	Deceased
Follow-up, months	19	18	16	2

Mean WBC, ESR, and CRP values at presentation were 6.8 x 10^3^/μL (range: 6.22 x 10^3^/μL-7.46 x 10^3^/μL), 102.5 mm/1^st ^hour (range:72-123 mm/1^st^ hour), and 11.5 mg/dL (range: 2.96-23.8 mg/dL) respectively (normal range - WBC: 4-11 x 10^3^/μL; ESR: 0-20 mm/1^st ^hour; CRP: <0.8 mg/dL). The mean duration from arthroplasty to PJI diagnosis was 18.5 days (range: 12-25 days). *A. baumannii *PJI was isolated from culture samples collected from joint synovial fluid, deep tissue cultures, and/or the implant itself (Table 1).

**Figure 1:**
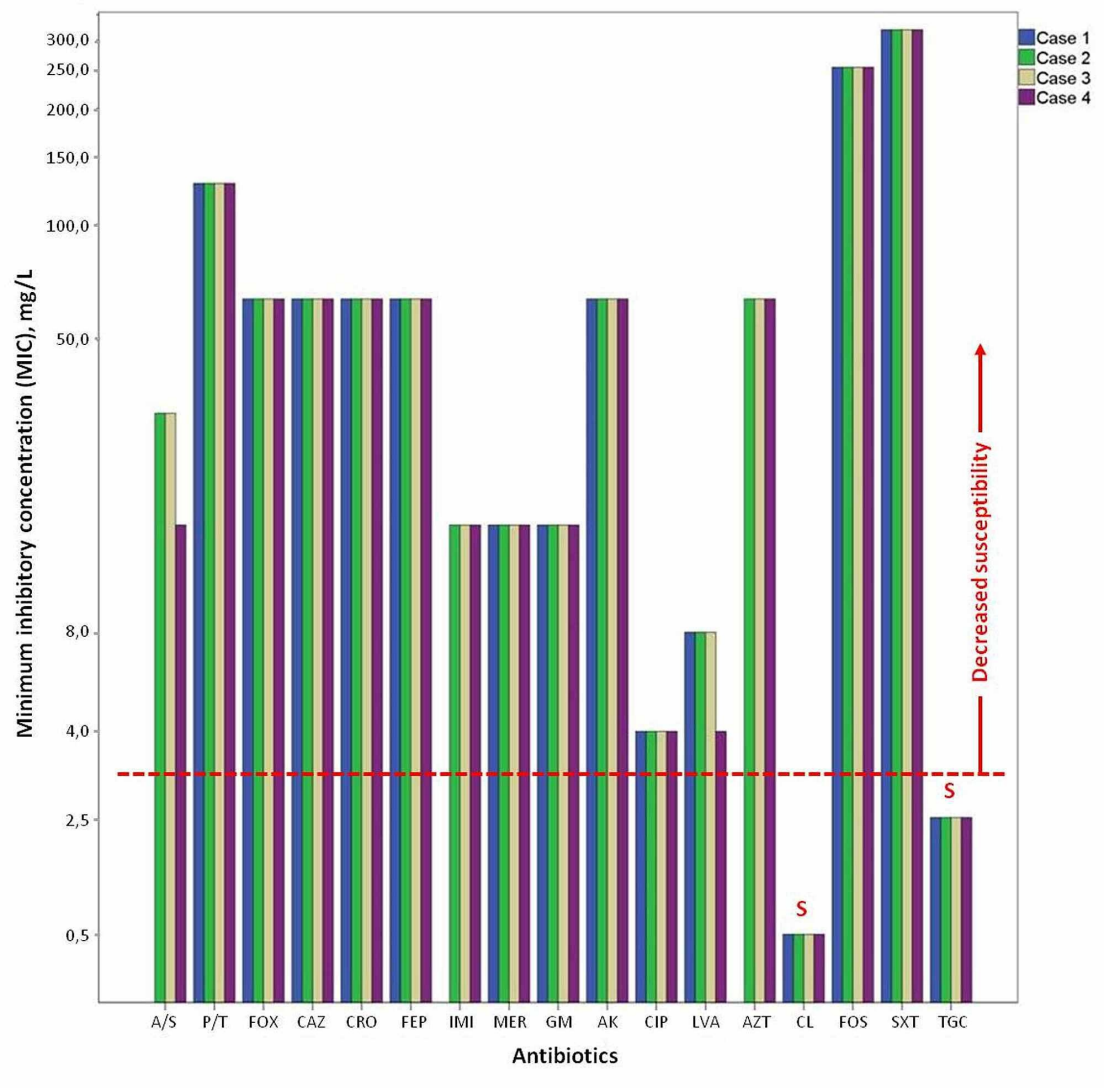
Antibacterial susceptibility expressed as a minimum inhibitory concentration (MIC), mg/L, of Acinetobacter baumannii to 17 antibiotics A/S: ampicillin/sulbactam; P/T: piperacillin/tazobactam; FOX: cefoxitin; CAZ: ceftazidime; CRO: ceftriaxone; FEP: cefepime; IMI: imipenem; MER: meropenem; GM: gentamicin; AK: amikacin; CIP: ciprofloxacin; LVA: levofloxacin; AZT: aztreonam; CL: colistin; FOS: fosfomycin; SXT: trimethoprim/sulfamethoxazole; TGC: tigecycline; S: sensitive

All patients included in the study were treated with the DAIR procedure. Surgical debridement was performed through the previous incision and included thorough irrigation of the joint cavity, sample collection for microbiological cultures (at least five) using separate sterile instruments, and modular exchange. The wound and the joint cavity were pulse lavage-irrigated using 6 liters of normal saline. Vancomycin (1 g intravenous every 12 hours) was the initial empirical antibiotic therapy, guided by our local prevalence of pathogens and their drug susceptibility patterns. All cultures were positive for *A. baumannii*. The antibacterial resistance of *A. baumannii* strains was determined for 17 antibiotics (Figure 1). *A. baumannii strains* displayed very high minimum inhibitory concentration (MIC) values for at least three classes of antibacterial agents, all penicillins-cephalosporins, fluoroquinolones, and aminoglycosides, thereby defining *A. baumannii* as MDR [10]. Thereafter, tigecycline (100 mg every 12 hours) and colistin (initial dose of 9 million IU followed by 4.5 million IU every 12 hours) were administrated intravenously for two to four weeks and subsequently switched to oral antibiotic therapy for at least three months. Antibiotic treatment was discontinued once clinical resolution and normalization of serum inflammatory markers were achieved. Laboratory monitoring (CRP, ESR, WBC counts, serum creatinine, and liver enzymes) was performed to assess the effectiveness of the antibiotic therapy and to detect any adverse events or potential toxicity.

The mean interval between arthroplasty and debridement was 25.5 days (range: 20-33 days). The mean intravenous antibiotic duration was 23.5 days (range: 20-28 days). Treatment was unsuccessful in two patients; one patient had a recurrence of the infection that required a second debridement and resection arthroplasty. Patient number 4 underwent DAIR, a resection arthroplasty, and died approximately two months after initial surgical treatment. The average duration of the follow-up for the three patients who survived was 17.66 months (range: 16-19 months) (Table 1).

## Discussion

Our approach to treating MDR *A. baumannii* PJI with the DAIR procedure was only partially successful in the present study since the infection was eradicated in only two out of four patients included. The genus *Acinetobacter* (originating from the Greek word “*akinetos*”, which means non-motile), which was first described in 1911, comprises aerobic gram-negative bacteria that can cause severe wound infections. An increasing number of infections caused by this microorganism has been recorded over the last three decades. Meanwhile, there has been a dramatic increase of antibiotic-resistant strains of the pathogen, limiting antibiotic treatment options for infection and posing a challenge to clinicians [11].

*A. baumannii* is an opportunistic nosocomial pathogen, rare in orthopaedic practice, which is associated with serious infections with high rates of morbidity and mortality. PJI caused by *Acinetobacter* species is relatively rare, with an incidence ranging from 0.6% to 1.2% in the literature. Surprisingly, in our department, we encountered four cases of *A.* *baumannii* PJI within a one-year period. The high proportion of PJIs due to *A. baumannii*, compared to other pathogens, in our study contrasts with the data from other large epidemiological studies [6,7,11]. This could be attributed to contamination by *A. baumannii* of the surfaces in hospital rooms, such as beds, tables, sinks, medical devices, floor, and walls. Indeed, following the cleaning and disinfection of our medical wards, once the fourth case described in the present study was confirmed, the cases of *A. baumannii* PJIs were reduced to zero the following year. Contaminated environmental surfaces in hospital rooms can contribute to the transmission of hospital pathogens, including methicillin-resistant *Staphylococcus aureus*, vancomycin-resistant *Enterococcus* species, *Clostridium difficile*, *Acinetobacter* species, and norovirus [12]. Educational and training programs for healthcare workers and the development of new protocols and checklists regarding hand hygiene and surface disinfection could reduce environmental contamination and thereby the rate of hospital-acquired infections.

PJI is essentially a surgical disease and the majority of the cases require some form of surgical intervention. The DAIR remains a key element in the management of PJI, especially when the usual pathogens are involved, such as *Enterococcus*, *Staphylococcus,* and *Streptococcus* [13]. For acute PJIs caused by gram-negative bacteria and managed with the DAIR procedure, the prognosis is relatively good. Martínez-Pastor et al. [14] report a success rate of 74.5% for the treatment of gram-negative infections with debridement, implant retention, and antibiotic treatment. Interestingly, they found a positive correlation between favorable outcomes and CRP concentration below 15 mg/dl at the time of diagnosis and treatment with fluoroquinolones. In our study, we believe that the outcome of DAIR was affected by a number of factors, such as the advanced age of the patients, the comorbidities, the time interval between arthroplasty and DAIR, and the presence of the specific bacterial pathogen, which were in a complex interplay.

Currently, there is no consensus regarding the real efficacy of the DAIR procedure. Previous studies have shown variable treatment success rates ranging from 31% to 100% for acute infections. Many factors may have contributed to this wide range of reported success rates. The type of infection, the number and species of pathogens isolated, the time of the initiation of treatment, and patient characteristics are of great importance for a successful clinical outcome [4]. It is reported that joint infection caused by MDR *Acinetobacter* is associated with higher failure rates and, hence, prosthesis retention is not recommended. The production of a mature biofilm, which evades the host defense, causes a delay in the clinical presentation and leads to failure of the antibiotic treatment due to the lack of a systemic response and is one of the main reasons proposed for DAIR failure [3].

In a recent study, Kazimoglou et al. [15] reported a limited success rate (41%) for the DAIR procedure for the treatment of seven patients with early-onset hemiarthroplasty infections caused by either gram-positive or gram-negative bacteria. They also found that successful outcomes are positively correlated with a duration of the infection after implantation under two weeks and a sedimentation value lower than 60 mm/h. However, It should be noted that, in their study, modular components of the prosthesis were not exchanged, which could be the reason for the poor outcomes. On the other hand, Sukeik et al. [16] advocate aggressive irrigation and exchange of modular parts for both early and late infections with a short duration of symptoms, and they achieved a 100% success rate.

The DAIR procedure may not be a viable treatment option once a mature biofilm is formed around the prosthesis. The DAIR is not recommended if the interval between the onset of the infection and the debridement is more than four weeks. Complete removal of the prosthesis is necessary for patients with a longer duration of symptoms and when a mature biofilm is formed around the implant [3,11]. The establishment of a biofilm around a prosthesis contributes to the extensive dissemination of MDR strains of gram-negative bacteria. *A. baumannii*, a gram-negative microorganism, is capable of forming a biofilm, which serves as a physical barrier to the diffusion of antibacterial agents, rendering it even more resistant [15,17].

Previous studies suggest excellent treatment outcomes for PJIs caused by gram-negative bacteria, susceptible to fluoroquinolones [17,18]. Fluoroquinolones demonstrate not only a favorable pharmacokinetic profile but also an excellent penetration in gram-negative biofilms. However, the increasing fluoroquinolone-resistance rates among gram-negative bacteria and the emergence of MDR *Acinetobacter* infections have limited their use in PJIs. In our study, the isolated *A. Baumannii* was resistant to the commonly recommended antibiotics for PJIs in all cases. A combination therapy involving tigecycline and colistin has been shown to decrease bacterial counts, prevent bacterial regrowth at subinhibitory concentrations, and lead to promising results in the treatment of complex PJIs [19,20]. Vila et al. [19], in a cases series of three patients with *Acinetobacter* PJI who were treated with the DAIR and a combination of high-dose tigecycline and colistin in the immediate postoperative period, reported a 100% success rate, with none of the patients showing any sign of relapse in the follow-up period.

## Conclusions

In conclusion, this study presented a case series of PJI due to MDR *A. baumannii* managed with the DAIR procedure. Our results show a 50% success rate in fluoroquinolone-resistant, gram-negative bacteria treated with debridement, implant retention, and a combination of tigecycline and colistin antibiotic therapy. The DAIR procedure resulted in a limited success rate for the management of MDR *A. baumannii *PJIs. MDR *A. baumannii* PJI is more challenging to be eradicated compared to infections caused by common gram-positive pathogens. Further long-term studies, with a larger number of patients, are required to validate our results.
